# The HPB-AML-I cell line possesses the properties of mesenchymal stem cells

**DOI:** 10.1186/1756-9966-29-163

**Published:** 2010-12-13

**Authors:** Bambang Ardianto, Takeshi Sugimoto, Seiji Kawano, Shimpei Kasagi, Siti NA Jauharoh, Chiyo Kurimoto, Eiji Tatsumi, Keiko Morikawa, Shunichi Kumagai, Yoshitake Hayashi

**Affiliations:** 1 Division of Molecular Medicine and Medical Genetics, Department of Pathology, Graduate School of Medicine, Kobe University, Kobe, Japan; 2 Department of Clinical Pathology and Immunology, Graduate School of Medicine, Kobe University, Chuo-Ku, Kobe 650-0017, Japan; 3 Division of Clinical Nutrition, Department of Nutrition, Sagami Women's University, Sagamihara, Japan

## Abstract

**Background:**

In spite of its establishment from the peripheral blood of a case with acute myeloid leukemia (AML)-M1, HPB-AML-I shows plastic adherence with spindle-like morphology. In addition, lipid droplets can be induced in HPB-AML-I cells by methylisobutylxanthine, hydrocortisone, and indomethacin. These findings suggest that HPB-AML-I is similar to mesenchymal stem cells (MSCs) or mesenchymal stromal cells rather than to hematopoietic cells.

**Methods:**

To examine this possibility, we characterized HPB-AML-I by performing cytochemical, cytogenetic, and phenotypic analyses, induction of differentiation toward mesenchymal lineage cells, and mixed lymphocyte culture analysis.

**Results:**

HPB-AML-I proved to be negative for myeloperoxidase, while surface antigen analysis disclosed that it was positive for MSC-related antigens, such as CD29, CD44, CD55, CD59, and CD73, but not for CD14, CD19, CD34, CD45, CD90, CD105, CD117, and HLA-DR. Karyotypic analysis showed the presence of complicated abnormalities, but no reciprocal translocations typically detected in AML cases. Following the induction of differentiation toward adipocytes, chondrocytes, and osteocytes, HPB-AML-I cells showed, in conjunction with extracellular matrix formation, lipid accumulation, proteoglycan synthesis, and alkaline phosphatase expression. Mixed lymphocyte culture demonstrated that CD3^+ ^T-cell proliferation was suppressed in the presence of HPB-AML-I cells.

**Conclusions:**

We conclude that HPB-AML-I cells appear to be unique neoplastic cells, which may be derived from MSCs, but are not hematopoietic progenitor cells.

## Background

Mesenchymal stem cells (MSCs) constitute a cell population, which features self-renewal and differentiation into adipocytes, chondrocytes, and osteocytes. Human MSCs have been isolated from various tissues and organs, such as muscle, cartilage, synovium, dental pulp, bone marrow, tonsils, adipose tissues, placenta, umbilical cord, and thymus (reviewed by [1]). The biological roles of MSCs were initially described by Friedenstein and colleagues in 1970s. They observed bone formation and reconstitution of the hematopoietic microenvironment in rodents with subcutaneously transplanted MSCs (reviewed by [2]). In addition to providing support for the early stage of hematopoiesis, MSCs have also been reported to suppress the proliferation of CD3^+ ^T-cells [3], which led to the utilization of MSCs in the management of various pathologic conditions, such as graft-versus-host disease (GvHD) after allogeneic bone marrow transplantation (reviewed by [4-6]). Recent studies have successfully isolated cancer-initiating cells with properties similar to those of MSCs from cases with some neoplasms, such as osteosarcoma [7], Ewing's sarcoma [8], and chondrosarcoma [9]. Furthermore, the characteristics of MSCs isolated from cases with hematopoietic neoplasms have also been investigated. Shalapour *et al. *[10] and Menendez *et al. *[11] identified the presence of oncogenic fusion transcripts, such as *TEL*-*AML1*, *E2A*-*PBX1*, and *MLL *rearrangements, in MSCs isolated from cases with B-lineage acute lymphoblastic leukemia (B-ALL). These reports suggested that some leukemias may be derived from the common precursors of both MSCs and hematopoietic stem cells (HSCs).

HPB-AML-I has been considered a unique cell line. In spite of its establishment from the peripheral blood mononuclear cells (PBMCs) of a case with acute myeloid leukemia (AML)-M1, this cell line reportedly has the features of spindle-like morphology and plastic adherence [12]. The detached HPB-AML-I cells were surprisingly capable of proliferating and adhering to plastic surfaces after passage. Immunophenotypic analysis of HPB-AML-I demonstrated the absence of hematopoietic cell-surface antigens and showed that this cell line resembles marrow stromal cells [12]. Moreover, in the presence of methylisobutylxanthine, hydrocortisone, and indomethacin, but not troglitazone, an increase in the number of lipid droplets was observed in these cells [12]. In view of these features, we further investigated the possibility of HPB-AML-I being a neoplasm of MSC origin.

Recently, some human MSC lines have been established from the bone marrow [13,14] and umbilical cord blood [15] cells of healthy donors. To establish a stable cell line, genes encoding the human telomerase reverse transcriptase (hTERT), bmi-1, E6, and E7 proteins were transduced to prolong the life span of the healthy donor-originated MSCs [13-15]. However, there have been no reports of the establishment of MSC lines from human bone marrow cells without *in vitro *gene transduction. Since a number of the characteristics of HPB-AML-I are not typically observed in leukemic cells, we wondered whether HPB-AML-I cells are neoplastic cells originating from the non-hematopoietic compartments of bone marrow, such as MSCs.

## Methods

### Cell lines and cell culture

HPB-AML-I cells were kindly provided by Dr. K. Morikawa (Sagami Women's University, Sagamihara, Japan) and 5 × 10^5 ^of these cells were cultured in 10 ml of RPMI-1640 medium supplemented with L-glutamine (Gibco, Carlsbad, CA), 10% fetal bovine serum (FBS) (Clontech, Mountain View, CA), 50 U/ml of penicillin (Lonza, Walkersville, MD), and 50 μg/ml of streptomycin (Lonza). Cell culture was performed in a T-25 flask and was maintained in a 37°C incubator humidified with 5% CO_2_. Cell passage was performed twice a week. UCBTERT-21, the *hTERT*-transduced umbilical cord blood mesenchymal stem cell (MSC) line [15], was obtained from the Japanese Collection of Research Bioresources (JCRB, Osaka, Japan) and propagated in a T-75 flask in a total number of 1.5 × 10^5 ^cells. Cell culture was maintained in 15 ml of Plusoid-M medium (Med Shirotori, Tokyo, Japan) containing 5 μg/ml of gentamicin (Gibco). The culture medium was replaced twice a week and cell passage was performed when the cultured cells reached 80-90% of confluence.

### Cytochemical analysis

The following cytochemical staining was performed according to the manufacturer's instructions: May Grünwald-Giemsa (Sysmex, Kobe, Japan), myeloperoxidase-Giemsa, toluidine blue, alkaline phosphatase-Safranin O (Muto, Tokyo, Japan), Sudan Black B-hematoxylin, oil red O-hematoxylin (Sigma-Aldrich, St. Louis, MO), and von Kossa-nuclear fast red (Diagnostic BioSystems, Pleasanton, CA).

### Cytogenetic analysis

Cytogenetic analysis was performed according to the standard protocols. The karyotype was determined by G-banding using trypsin and Giemsa (GTG) [16] to examine 50 cells. The best metaphase was then photographed to determine the karyotype. The specimen was also submitted to spectral karyotyping (SKY)-fluorescence *in situ *hybridization (FISH) assay according to Ried's method using whole chromosome painting (WCP) libraries (cytocell for WCP) and α-satellite DNA probes [17].

### Cell-surface antigen analysis

Flow cytometric analysis was performed by using the following monoclonal antibodies recommended by the International Society for Cellular Therapy (ISCT) (reviewed by [2]) and monoclonal antibodies used in the study of Wang *et al. *[18]: MφP9 (CD14), SJ25C1 (CD19), MAR4 (CD29), 8G12 (CD34), 515 (CD44), 2D1 (CD45), IA10 (CD55), p282 (CD59), AD2 (CD73), 5E10 (CD90), SN6 (CD105), 104D2 (CD117), and L243 (HLA-DR). All of these monoclonal antibodies were obtained from BD Biosciences (San Jose, CA), except for SN6 from Invitrogen (Carlsbad, CA). Cells were resuspended in a total number of 2 × 10^5 ^in 50 μl of phosphate-buffered saline (PBS) supplemented with 4% FBS, then incubated with 20 μl of monoclonal antibodies, except for 5E10 (2 μl) and SN6 (5 μl), for 45 min at 4°C, and the conjugated cells fixed with 1 ml of 4% paraformaldehyde solution (Wako, Osaka, Japan). Flow cytometric analysis was performed with Cell Quest software and the FACSCalibur device (BD Biosciences) to examine 20,000 events.

### *In vitro *differentiation toward adipocytes, chondrocytes, and osteocytes

To induce adipogenesis and osteogenesis, 1 × 10^3 ^cells were cultured in 500 μl of medium in a four-well chamber slide. Three days after propagation, the culture medium was replaced with 500 μl of StemPro adipogenesis or osteogenesis differentiation medium (Gibco) containing 5 μg/ml of gentamicin. Chondrogenesis was induced with a micromass culture system [19,20], in which 5 × 10^2 ^of the cells were resuspended in 10 μl of culture medium and applied to the center of a culture well. A 96-well culture plate was used in our study. Two hours after propagation, 100 μl of StemPro chondrogenesis differentiation medium containing 5 μg/ml of gentamicin was added. The differentiation medium was replaced twice a week.

### Mixed lymphocyte culture assay

PBMCs were separated from the heparinized peripheral blood of a healthy donor by means of Ficoll-Paque density gradient centrifugation (Amersham Biosciences, Uppsala, Sweden). CD3^+ ^T-cells were purified from PBMCs by magnetic-activated cell sorting (MACS) positive selection (Miltenyi Biotec, Auburn, CA) and 1 × 10^6 ^of these cells were cultured for 48 h in a 96-well culture plate in the presence of 12.5 μg/ml of phytohemagglutinin (Wako) with or without irradiated (25 Gy) HPB-AML-I and UCBTERT-21 (0, 1 × 10^3^, 1 × 10^4^, and 1 × 10^5 ^cells/well) cells. From each culture well, 100 μl of cell suspension was pulsed with 10 μl of Cell Counting Kit-8 solution (Dojindo, Tokyo, Japan) at 37°C for 4 h. The optical density at 450 nm was measured to determine cell viability in each of the culture wells.

## Results

### HPB-AML-I shows plastic adherence, negative myeloperoxidase expression, and complex chromosomal abnormalities

**Figure 1:**
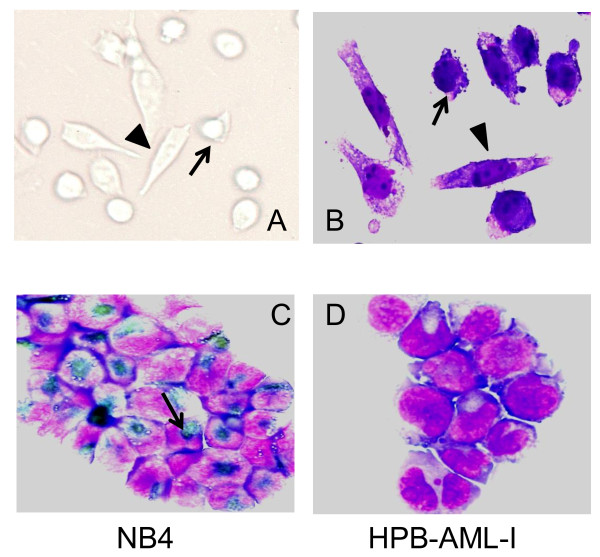
**Morphological and cytochemical characteristics of HPB-AML-I**. Inverted microscopic examination (A) and May Grünwald-Giemsa staining (B) revealed that HPB-AML-I features a round-polygonal (arrow) and spindle-like (arrowhead) morphology. The human acute promyelocytic leukemia (APL) NB4 cell line was used as positive control for myeloperoxidase staining. Positive reactions are indicated with an arrow (C). Absence of myeloperoxidase expression was observed in the cytospin-prepared HPB-AML-I cells (D). Original magnification ×400.

Inverted microscopic examination (Figure 1A) and May Grünwald-Giemsa staining (Figure 1B) of HPB-AML-I cells revealed that this cell line is composed of round-polygonal and spindle-like cells. Unlike the round-polygonal cells, HPB-AML-I cells with the spindle-like morphology attached to plastic surfaces. Since HPB-AML-I was established from a case with AML, we examined this cell line for the presence of myeloperoxidase expression. The human acute promyelocytic leukemia (APL) NB4 cell line was used as positive control in this examination (Figure 1C). We found that HPB-AML-I was negative for myeloperoxidase expression (Figure 1D).

**Figure 2:**
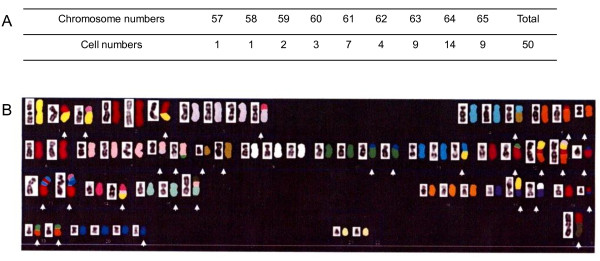
**Cytogenetic features of HPB-AML-I**. Karyotypic analysis performed on 50 HPB-AML-I cells demonstrated that each of these cells had abnormal chromosome numbers ranging from 57 to 65 (modal: 64) (A). Reverse DAP (left side) and SKY-FISH (right side) of a representative HPB-AML-I cell with a total number of 64 chromosomes are shown. The complete karyotype has been reported as: 61-65 <3n>, X, -X, -Y, der(X) t(X;2)(p22.1;?), der(1;18)(q10;q10), der(1;22)(q10;q10), der(2) (2pter→2q11.2::2?::1p21→1pter), +der(3) t(3;14)(p13;q?), der(4) t(4;8)(q11;q11.2), der(5) t(5;18)(p13;p11.2), i(5)(p10), -6, +der(7) t(3;7)(?;q11.2), +der(7) t(7;19)(q22;q13.1), -8, der(8) del(8)(p?) del(8)(q?), der(8) (qter→q22::p23→qter), -9, +10, der(10;20)(q10;q10)x2, der(11) t(1;11)(?;q13), der(12) t(12;19)(p13;q13.1), +der(12) (5qter→5q13::12?::cen::12?::1?), +der(12) (5qter→5q13::12?::cen::12?::1?::3?), -13, der(13) (13qter→13p11.2::11?::13?::11?), der(13) (13qter→13p11.2::11?::20?::11?::22?), -14, der(14) (14pter→14q24::3?::1?), der(15) (15?::p11.2→q13::q15→qter), der(15) (15qter→15p11.2::7?::X?), -16, der(17) t(1;17)(p13;p11.2), der(17) t(9;17)(?;p11.2), der(18) t(18;?)(q11.2;?), -19, der(19) t(5;19)(?;q11), +20, +20, +der(20) t(17;20)(?;p11.2), -21, -22, -22, +der(?) t(?;12)(q;15) (B).

HPB-AML-I was also subjected to cytogenetic analysis, which demonstrated the presence of a complex karyotype with a modal chromosome number of 64 (range: 57-65; Figure 2A). A single X chromosome and a number of other abnormalities, mainly consisting of chromosome gains, chromosome losses, translocations, and deletions, were detected by SKY-FISH assay (Figure 2B). There were no reciprocal chromosomal translocations, which are frequently observed in AML cases.

### HPB-AML-I expresses cell-surface antigens characteristic for MSCs

**Figure 3:**
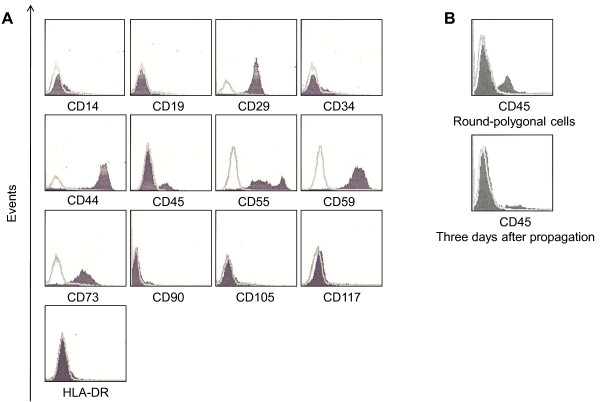
**Phenotypic profiles of HPB-AML-I**. The expression of MSC-related antigens in the HPB-AML-I cell line is shown (A). CD45 expression of round-polygonal HPB-AML-I cells (upper) and of the cells, which were cultivated for three days after propagation of round-polygonal HPB-AML-I cells (lower), are shown (B). Flow cytometric results for the antigens indicated are shown in black. IgG κ isotype (not shaded) was used as negative control.

HPB-AML-I was examined by means of flow cytometric analysis for cell-surface antigens, which are widely used to identify the presence of MSCs. HPB-AML-I expressed CD29, CD44, CD55, CD59, and CD73, but no cell-surface expression of CD14, CD19, CD34, CD90, CD105, CD117, or HLA-DR was detected (Figure 3A). The cell-surface antigen expression patterns of UCBTERT-21 [15] and F6 [21] cell lines and human MSCs isolated from aorta-gonad-mesonephros, yolk sac [18], bone marrow [22], and umbilical cord blood [23] are presented in Table 1 for comparison, showing that there are phenotypic similarities between HPB-AML-I and UCBTERT-21, which was established from human umbilical cord blood and transduced with *hTERT*.

**Table 1:** Cell-surface antigen expression in HPB-AML-I and other MSCs

Antigens	HPB-AML-I	**UCBTERT-21 **[15]	**F6 **[21]	**ISCT criteria **[2]	**Wang *et al. ***[18]	**Lee *et al. ***[22]	**Majore *et al. ***[23]
CD14	-	-	-	-	-	-	ND
CD19	-	ND	ND	-	-	ND	ND
CD29	+	+	+	ND	+	ND	ND
CD34	-	-	-	-	-	ND	ND
CD44	+	+	+	ND	+	+	+
CD45	-	-	-	-	-	ND	ND
CD55	+	+	ND	ND	ND	ND	ND
CD59	+	+	ND	ND	ND	ND	ND
CD73	+	ND	ND	+	+	ND	+
CD90	-	-	ND	+	ND	+	+
CD105	-	ND	ND	+	+	+	+
CD117	-	-	ND	ND	ND	ND	ND
HLA-DR	-	ND	-	-	-	ND	ND

ND: not determined

Flow cytometric analysis showed that 11.9% of HPB-AML-I cells expressed CD45 (Figure 3A). We postulated that the presence of two morphological phases of HPB-AML-I cell line may be related to CD45 expression. For addressing this hypothesis, we performed a prolonged cell culture to increase the confluence, resulting in a morphological change of spindle-like HPB-AML-I cells toward round-polygonal. The round-polygonal cells, which were harvested from a confluent culture with gently washing, but no trypsinization, were positive for CD45 in 25.7% of cells (Figure 3B). Interestingly, the CD45 expression returned to low positivity (10.1%) after the round-polygonal cells were cultivated for another three days, when they became adherent and spindle-like (Figure 3B).

### HPB-AML-I cells are capable of acquiring the properties of adipocytes, chondrocytes, and osteocytes

**Figure 4:**
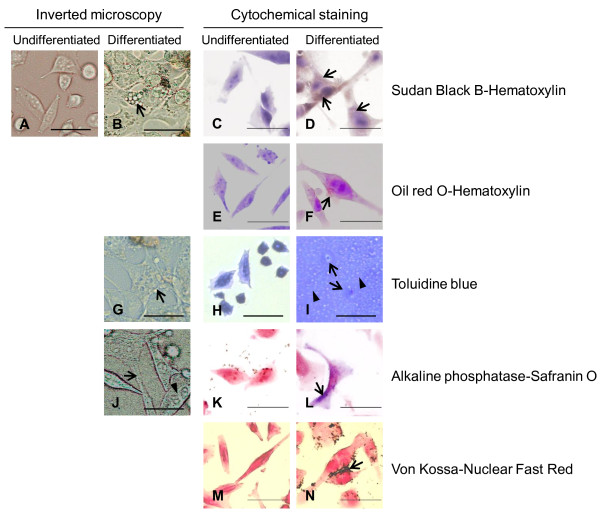
**Morphological and cytochemical changes in HPB-AML-I cells following the induction of differentiation toward mesenchymal lineage cells**. Undifferentiated HPB-AML-I cells observed with an inverted microscope are shown for comparison (A). A representative HPB-AML-I cell induced to differentiate toward adipocyte and showing spindle-like morphology and cytoplasmic vacuoles is indicated with an arrow (B). Undifferentiated (C, E) and differentiated (D, F) HPB-AML-I cells were stained with Sudan Black B (C, D) and oil red O (E, F). The nucleus was counterstained with hematoxylin. Positive Sudan Black B and oil red O staining of cytoplasmic vacuoles of the differentiated HPB-AML-I cells is indicated with an arrow. Following the induction of differentiation toward chondrocytes, HPB-AML-I cells showed polygonal morphology with a number of cytoplasmic vacuoles (arrow) (G). The micromass of undifferentiated (H) and differentiated (I) HPB-AML-I cells were stained with toluidine blue. The presence of lacunae (arrows) and the toluidine blue-positive extracellular matrix (arrowheads) characteristic for a cartilage were observed following the induction of chondrogenesis. The osteogenic-differentiated HPB-AML-I cells demonstrated a number of cell processes (arrow) and an eccentrically located nucleus (arrowhead) (J). Undifferentiated (K) and differentiated (L) HPB-AML-I cells were cytochemically examined for alkaline phosphatase expression. The nucleus was counterstained with Safranin O. Positive reactions are shown in the differentiated HPB-AML-I cells with an arrow. Undifferentiated (M) and differentiated (N) HPB-AML-I cells were stained with von Kossa method. The nucleus was counterstained with nuclear fast red. The extracellular depositions of calcium following the induction of osteogenesis are indicated with an arrow. Original magnification x400; Size bar: 20 μm.

To investigate the multipotency of HPB-AML-I cells, we induced them to differentiate toward adipocytes, chondrocytes, and osteocytes. For comparison, the results of examination of undifferentiated HPB-AML-I cells with an inverted microscope are also shown (Figure 4A). Two weeks after the induction of adipogenesis, morphological changes were observed in HPB-AML-I cells. The differentiated cells retained the spindle-like morphology or appeared as large polygonal cells. In addition, cytoplasmic vacuoles of various sizes were observed and inverted microscopic examination showed that these vacuoles occurred in solitary or aggregated formations (Figure 4B). While Sudan Black B and oil red O did not stain the cytoplasm of undifferentiated cells (Figure 4C and 4E), the cytoplasmic vacuoles of differentiated HPB-AML-I cells were positive for these cytochemical staining (Figure 4D and 4F), suggesting the presence of lipid accumulation in the adipogenic-differentiated HPB-AML-I cells.

Two weeks after the induction of chondrogenesis, the differentiated HPB-AML-I cells showed polygonal morphology, which made them distinct from the undifferentiated cells. Inverted microscopic examination demonstrated the presence of a number of vacuoles in the cytoplasm of differentiated HPB-AML-I cells (Figure 4G). In contrast to the undifferentiated cells (Figure 4H), the differentiated HPB-AML-I cells formed lacunae. The proteoglycan-rich extracellular matrix, as indicated by positive toluidine blue staining, surrounded the lacunae (Figure 4I). The presence of lacunae, as well as extracellular proteoglycan accumulation, suggested that the micromass of chondrogenic-differentiated HPB-AML-I cells acquires the properties of a cartilage.

Inverted microscopic examination three weeks after the induction of osteogenesis demonstrated the presence of a number of cell processes and an eccentrically located nucleus in the differentiated HPB-AML-I cells (Figure 4J). The undifferentiated cells did not express alkaline phosphatase as shown by negative cytochemical staining for this protein (Figure 4K). On the other hand, cytochemical staining resulted in positive staining for alkaline phosphatase in the cytoplasm of differentiated HPB-AML-I cells (Figure 4L). Moreover, the differentiated HPB-AML-I cells also secreted calcium, which constitutes the extracellular matrix of the bone, as shown by von Kossa staining (Figure 4M and 4N). These two findings suggested the acquisition of osteogenic characteristics by HPB-AML-I cells following the induction of osteogenesis.

### Inhibition of CD3^+ ^T-cell proliferation in the presence of HPB-AML-I cells

**Figure 5:**
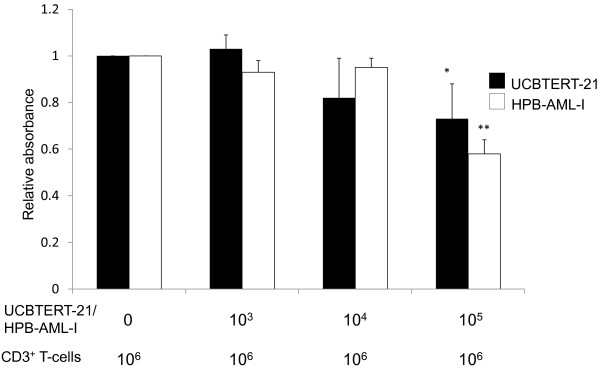
**Inhibition of CD3^+ ^T-cell proliferation in the presence of HPB-AML-I cells**. Mixed lymphocyte culture was performed in the presence or absence of HPB-AML-I cells (white columns). For control, similar experiments were performed with UCBTERT-21 cells (black columns). Results are presented as the XTT absorbance levels at 450 nm, which were normalized to those of the baseline experiments (cell culture in the absence of HPB-AML-I or UCBTERT-21 cells). Means and standard deviations of four independent experiments are shown. *, *P *< 0.05; **, *P *< 0.01 compared to the baseline results

CD3^+ ^T-cells obtained from peripheral blood were cultured with or without HPB-AML-I cells. The XTT absorbance levels at 450 nm, which show the viability of CD3^+ ^T-cells, decreased in a dose-dependent manner similar to those of UCBTERT-21 (Figure 5). These findings suggested that HPB-AML-I cells dose-dependently suppress the antigen-driven proliferation of CD3^+ ^T-cells, which is also characteristic of MSCs.

## Discussion

Even though HPB-AML-I was established from the PBMCs of an AML-M1 case [12], this cell line presents distinctive morphological features from AML. In terms of cell-surface antigen expression, multilineage differentiation, and CD3^+ ^T-cell suppression, the characteristics of HPB-AML-I were found to be similar to those of MSCs. Our findings presented here suggest that HPB-AML-I may be a neoplastic cell line with MSC properties. Few reports have dealt with the establishment of human neoplastic MSC lines. A previous study established F6, a human neoplastic MSC line, from embryonic bone marrow MSCs. Transplantation of F6 cells into the SCID-nude mice resulted in fibrosarcoma formation and tissue metastasis [21,24]. To the best of our knowledge, however, HPB-AML-I is the first neoplastic MSC line derived from a leukemic case.

The appearance of HPB-AML-I cells in suspension phase with their round-polygonal morphology intrigued us. We observed that an increase in the population of HPB-AML-I cells with such morphological patterns occurs in conjunction with the increased confluence of cultured cells. Morphological changes during culturing have previously been described in the case of bone marrow MSCs. Choi *et al. *[25] reported that the morphology of bone marrow MSCs changed from small spindle-like in the first passage to large polygonal in the later passages. In contrast to many other adherent cell lines, HPB-AML-I cells with their round-polygonal morphology were viable and capable of proliferating and adhering to plastic surfaces following cell passage. Similar findings have been reported for the F6 cell line [21]. While the exact mechanisms remain to be elucidated, we speculate that the loss of adherent capacity after confluent condition may be a pivotal property to neoplasms originated from mesenchymal stem cells.

Flow cytometric analysis of HPB-AML-I disclosed that, based on ISCT criteria, the cell-surface antigen expression patterns of this cell line were similar to those of human MSCs (reviewed by [2]) with positive CD73 and negative CD14, CD19, CD34, CD45 and HLA-DR expression. However, contrary to those criteria (reviewed by [2]), HPB-AML-I did not express CD90 and CD105. Absence of CD90 expression has also been observed in UCBTERT-21 [15] and in human MSCs obtained from umbilical cord blood [15,26]. MSCs lacking CD105 expression have been reported by Jiang *et al. *[27] and Ishimura *et al. *[28], who isolated MSCs from the subcutaneous adipose tissue, and by Lopez-Villar *et al. *[29], who extracted MSCs from the bone marrow of a myelodysplastic syndrome case. These reports suggested that the absence of CD90 and CD105 expression in HPB-AML-I does not necessarily exclude the possibility that this cell line is derived from MSCs. The differentiation capability of MSCs with a negative CD105 expression has been investigated by Jiang *et al. *[27] and Ishimura *et al. *[28]. They found that this population of MSCs, while showing adipogenic differentiation, lacked chondrogenic and osteogenic differentiation. It is interesting that HPB-AML-I could differentiate into three lineages despite of CD105 negativity. In addition, a subpopulation of HPB-AML-I expressed CD45, even though most of HPB-AML-I cells were negative for CD45. Generally, CD45 is negative in MSCs, but CD45 expression has been detected in bone marrow MSCs from cases with multiple myeloma [30,31]. It is therefore not surprising that neoplastic MSC line, such as HPB-AML-I, shows the aberrant expression of this antigen. Interestingly, CD45 expression in HPB-AML-I cells is likely to be transient, as the expression levels of CD45 increased in round-polygonal cells in the confluent cell culture and they decreased after passage of round-polygonal cells. Normal cells are known to have the property of contact inhibition, which is lost in transformed cells. Therefore, cell-to-cell contact might induce the aberrant expression of CD45 with an unknown reason in HPB-AML-I cells.

By using inverted microscopic examination and cytochemical staining, we demonstrated that HPB-AML-I cells are able to acquire the properties of adipocytes, chondrocytes, and osteocytes. The capability of MSCs to differentiate toward mesenchymal lineage cells reportedly correlates with their morphological and cell-surface antigen expression patterns. Chang *et al. *[26] demonstrated that MSCs isolated from human umbilical cord blood consisted of cells with a flattened or spindle-like morphology and that the capability of differentiating toward adipocytes of the spindle-like MSCs was superior than that of the flattened cells. Since such heterogeneous morphology is shared by HPB-AML-I, further analyses are needed to characterize the difference between the round-polygonal and spindle-like cells.

As also reported by previous studies of the immunomodulatory effects on MSCs [18,32], we demonstrated that HPB-AML-I cells are capable of suppressing CD3^+ ^T-cell proliferation. Similar studies have been performed on MSCs isolated from cases with various hematopoietic neoplasms, such as ALL, Hodgkin's disease, non-Hodgkin's lymphoma, myelodysplastic syndrome, AML [33], and chronic myeloid leukemia (CML) [34]. In contrast to our results, Zhi-Gang *et al*. reported that bone marrow MSCs isolated from AML cases did not inhibit the proliferation of CD3^+ ^T-cells [33]. These findings suggest that bone marrow MSCs from cases with hematopoietic neoplasms may or may not be capable of inhibiting CD3^+ ^T-cell proliferation as a consequence of the secretion of humoral factors by neoplastic cells or the direct interaction with them. It is therefore very interesting that HPB-AML-I, regardless of its HSC or MSC origin, maintains the capability of inhibiting T-cell proliferation even after neoplastic transformation.

The cytogenetic analysis revealed the presence of complex chromosomal abnormalities in HPB-AML-I, although these were not the same as the frequently observed chromosomal alterations in AML cases. While it is not fully understood whether MSCs isolated from leukemic cases carry the cytogenetic characteristics common to leukemic cells, previous studies reported the absence of t(9;22)(q34;q11) chromosomal translocation or *BCR*-*ABL *rearrangement in bone marrow MSCs obtained from cases with Philadelphia (Ph) chromosome-positive CML [35,36]. On the other hand, a recent study demonstrated the presence of leukemic reciprocal translocation and fusion gene expression in bone marrow MSCs of *MLL*-*AF4*-positive B-ALL cases [11]. However, monoclonal Ig gene rearrangements, uncontrolled cell proliferation, diminished cell apoptosis, and cell-cycle arrest characteristic of leukemic cells were not observed in the bone marrow MSCs of those cases [11]. Unfortunately, we could not obtain the karyotype of the original leukemic cells. Therefore, the complex karyotype in HPB-AML-I may not correspond to the cytogenetic status of the primary cells. It is possible that the complex karyotype of HPB-AML-I may include the additional genetic changes, which occurred *in vitro *during and after the establishment of the cell line. Nevertheless, the MSC-like properties of HPB-AML-I, as shown in this study, suggest the possibility that the first genetic event might have occurred at the stage of MSC.

## Conclusions

In summary, we were able to demonstrate that HPB-AML-I has morphological, cytochemical, and phenotypic features, as well as the capability of differentiating toward mesenchymal lineage cells and of suppressing CD3^+ ^T-cell proliferation, which are all characteristic of MSCs. Our findings suggest that HPB-AML-I cells may represent a unique neoplastic cell line derived from bone marrow MSCs. We believe that this cell line will make an important contribution to a better understanding of the neoplastic transformation of bone marrow-derived constituents.

## List of abbreviations

ALL: acute lymphoblastic leukemia; AML: acute myeloid leukemia; APL: acute promyelocytic leukemia; CML: chronic myeloid leukemia; GvHD: graft-versus-host disease; FBS: fetal bovine serum; FISH: fluorescence *in situ *hybridization; GTG: G-banding using trypsin and Giemsa; HSC(s): hematopoietic stem cell(s); hTERT: human telomerase reverse transcriptase; ISCT: International Society for Cellular Therapy; MACS: magnetic-activated cell sorting, MSC(s): mesenchymal stem cell(s); PBMC(s): peripheral blood mononuclear cell(s); PBS: phosphate-buffered saline; SKY: spectral karyotyping; WCP: whole chromosome painting.

## Competing interests

The authors declare that they have no competing interests.

## Authors' contributions

BA, TS, and SK1 contributed to the experimental design, data acquisition and analyses, and manuscript preparation. SK2 contributed to the mixed lymphocyte culture analyses. SNAJ and CK contributed to the differentiation asssay. ET and KM contributed to the karyotypic analyses. SK3 and YH contributed to the data analysis and discussion. All authors read and approved the final manuscript.
